# E pluribus unum: Harmonization of physical functioning across intervention studies of middle-aged and older adults

**DOI:** 10.1371/journal.pone.0181746

**Published:** 2017-07-28

**Authors:** Nicole M. Armstrong, Laura N. Gitlin, Jeanine M. Parisi, Michelle C. Carlson, George W. Rebok, Alden L. Gross

**Affiliations:** 1 Department of Epidemiology, Johns Hopkins Bloomberg School of Public Health, Baltimore, Maryland, United States of America; 2 Johns Hopkins University Center on Aging and Health, Baltimore, Maryland, United States of America; 3 Division of Geriatrics and Gerontology, Department of Medicine, Johns Hopkins School of Medicine, Baltimore, Maryland, United States of America; 4 Center for Innovative Care in Aging, Johns Hopkins School of Nursing, Baltimore, Maryland, United States of America; 5 Department of Mental Health, Johns Hopkins Bloomberg School of Public Health, Baltimore, Maryland, United States of America; Universidad Europea de Madrid, SPAIN

## Abstract

Common scales for physical functioning are not directly comparable without harmonization techniques, complicating attempts to pool data across studies. Our aim was to provide a standardized metric for physical functioning in adults based on basic and instrumental activities of daily living scaled to NIH PROMIS norms. We provide an item bank to compare the difficulty of various physical functioning activities. We used item response theory methods to place 232 basic and instrumental activities of daily living questions, administered across eight intervention studies of middle-aged and older adults (N = 2,556), on a common metric. We compared the scale’s precision to an average z-score of items and evaluated criterion validity based on objective measures of physical functioning and Fried’s frailty criteria. Model-estimated item thresholds were widely distributed across the range of physical functioning. From test information plots, the lowest precision in each dataset was 0.80. Using power calculations, the sample size needed to detect 25% physical functional decline with 80% power based on the physical functioning factor was less than half of what would be needed using an average z-score. The physical functioning factor correlated in expected directions with objective measurements from the Timed Up and Go task, tandem balance, gait speed, chair stands, grip strength, and frailty status. Item-level harmonization enables direct comparison of physical functioning measures across existing and potentially future studies and across levels of function using a nationally representative metric. We identified key thresholds of physical functioning items in an item bank to facilitate clinical and epidemiologic decision-making.

## Introduction

Since 1950, over 100 standardized questionnaires of physical functioning ability have been developed for clinical and research use [1]. The scales are not easy to combine without techniques to harmonize, or link, them because each scale contains distinct questions which assess select dimensions of the broad range of physical functioning. Physical functioning refers to the ability to care for oneself, taking into account body activities dependent on both upper and lower extremities as well as the central body [2]. It is a reflection of overall health [3], and plays an important role in quality of life as well as clinical decision making [4]. In addition to direct observation, it can be measured by asking questions about difficulty with, level of dependence in, needing assistance with, or health limitations in performing basic activities of daily living (ADLs)[5] and instrumental activities of daily living (IADLs) [5, 6].

Prior research suggests physical functioning questions can be arranged along a continuum from basic ADLs to more complex IADLs [4, 6–19]. The NIH Patient Reported Outcomes Measurement Information System (PROMIS) follows this continuum notion and provides flexible, reliable measures of physical health as well as other constructs [2, 20].

The aim of this study is to provide a comprehensive physical functioning metric to achieve comparability across datasets for responses on questionnaires assessing ADLs and IADLs. This metric allows the comparison of results from disparate physical functioning scales on one common “ruler.” We evaluated the psychometric properties of the comprehensive metric of physical functioning factor, changes over time by study, and concurrent criterion validity by characterizing associations with performance-based measures of physical functioning. We further provided a means to evaluate the appropriateness of given items for populations with varying levels of physical functioning by supplying a table of item thresholds for all indicators used in our study.

## Materials and methods

### Participants

The sample consisted of 2,556 participants pooled from eight behavioral intervention trials: Advancing Better Living for Elders (ABLE, N = 319), Advancing Caregiver Training (ACT, N = 272), Alzheimer’s Quality of Life (ALZQOL, N = 88), Beat the Blues (BTB, N = 208), Care of Persons with Dementia in their Environments (COPE, N = 237), Resources for Enhancing Alzheimer’s Caregiver Health II (REACH II, N = 670), Tailored Activity Program (TAP, N = 60), and the Baltimore Experience Corps Trial (BECT, N = 702).

ABLE was a 15-month randomized controlled efficacy trial of a home-based intervention involving occupational and physical therapy home visits that provided a range of strategies including compensatory strategies, environmental modifications, and assistive devices designed to improve and/or ameliorate functional difficulties [21]. ACT was a 6-month randomized controlled efficacy trial of family caregivers of persons with dementia exhibiting behavioral symptoms that tested a home-based intervention to manage or reduce distressful behavioral symptoms [22]. ALZQOL was a study of community-living persons with dementia and their caregivers designed to examine potentially modifiable factors associated with quality of life [23]. BTB was a 9-month randomized trial of a home-based intervention designed to reduce depressive symptoms in older urban African Americans [24]. COPE was a randomized intervention to test a behavioral approach to supporting physical functioning and quality of life of persons with dementia and caregiver well-being [25]. REACH II was a multi-site randomized intervention to improve quality of caregiving among dementia caregivers [26]. The TAP study tested a home-based occupational therapy intervention designed to provide activities tailored to interests and abilities of people with dementia and train their family caregivers in their use as part of routine care [27]. The BECT was a 24-month randomized intervention to evaluate effects of volunteering in elementary schools on older adults’ physical, social, and cognitive outcomes [28].

Institutional Review Boards at each study’s home institution approved study procedures. Johns Hopkins Bloomberg School of Public Health approved the IRB application for this study. The approval number is IRB00005716. All procedures performed in studies involving human participants were in accordance with the ethical standards of the institutional and/or national research committee and with the 1964 Helsinki declaration and its later amendments or comparable ethical standards. This article does not contain any studies with animals performed by any of the authors. Informed consent was obtained from all individual participants included in the study. The study was approved by the Johns Hopkins Bloomberg School of Public Health Institutional Review Board.

### Indicators of physical functioning

We considered questions assessing ADLs and IADLs for inclusion as physical functioning indicators. Items came from assessments administered to participants or their caregivers in each of the datasets using various forms. COPE and ABLE administered a self-reported physical disability questionnaire [29]. All datasets except for BECT administered the Caregiver Assessment of Function and Upset, a 15-item measure of physical functioning dependence [30]. Mode of administration differed across datasets. In ABLE, BTB, and BECT, participants were asked directly about several ADLs and IADLs. In ACT, ALZQOL, TAP, COPE, and REACH II, caregivers were asked about ADLs and IADLs of the person with dementia. Not all items overlapped across all datasets (S1 Table).

### Statistical analysis

We first described basic demographic and health characteristics of the sample in each dataset. We then derived and validated the physical functioning factor in three steps.

#### Derivation and validation of physical functioning

First, we identified physical functioning questions in each dataset, and determined which items and questionnaires were in common using available codebooks and tabulations of variables in the datasets themselves. We empirically tested the equivalency of items across datasets by testing for differential item functioning using multiple indicator, multiple causes (MIMIC) models [31]. We identified anchor items for MIMIC models using the free baseline-designated anchor approach [32]. We evaluated the dimensionality of physical functioning questions using parallel analysis with scree plots [33, 34]. We used Cronbach’s alpha[35] to characterize the internal consistency of the questions in each dataset.

Next, we performed confirmatory factor analysis of available physical functioning indicators. We estimated a model equivalent to a 1-parameter logistic graded response item response theory model [36, 37] using polychoric correlations. We externally scaled the derived physical functioning scale to the NIH PROMIS Physical Function item bank (version 1.0) using 26 items in common between the questions among the eight behavioral intervention trials and NIH PROMIS. We did so by fixing item thresholds for items with analogs in NIH PROMIS to publicly available threshold parameters from NIH PROMIS (http://www.assessmentcenter.net), which places the metric of the latent variable on the scale of the NIH PROMIS normative sample (Wave 1, N = 5329) [38]. Factor scores from the model represent the physical functioning score, and were scaled so that higher scores indicate lower physical functioning or greater disability. We used an expectation-maximization algorithm for maximum likelihood estimation with robust standard errors for the model in Mplus v7.11 [39].

#### Precision

To evaluate precision of the measurement model over a range of possible physical functioning ability scores for each dataset, we plotted test information curves [40]. To compare precision of the physical functioning factor score with a naïve average of available items, in the BECT dataset we calculated the sample size needed to detect change in physical functioning with 80% power using the physical functioning factor, and compared it against a naïve average score of items available in the datasets [41].

#### Validity

We evaluated face validity of the physical functioning factor by comparing model-estimated changes in physical functioning since enrollment among datasets with more than two study waves spanning at least six months (ABLE, ACT, BTB, TAP, and BECT).

We evaluated convergent criterion validity with associations of the physical functioning factor with available objective measures of physical functioning and Fried’s frailty criteria [42] in two of the eight datasets, overall and stratified by sex and age. We used the Timed Up and Go Task in ALZQOL, categorized into poor mobility (21+ seconds) and normal mobility (20 or fewer seconds). In BECT, we used four objective measures of lower and upper body objective physical performance: tandem balance (able to stand with tandem footing for 10+ seconds vs. unable), gait speed (faster vs. slower than 0.8 m/s), chair stands (faster vs. slower than the sample median), and grip strength (stronger vs. weaker than the sample median). Also, in BECT, we used Fried’s frailty criteria to define frail, pre-frail, and robust status [42]. We compared mean physical functioning across each group defined by the objective measure using linear regressions adjusted for age and sex.

#### Simulation

Given the different sets of physical functioning questions in each study, we evaluated the quality of the linkage between the nationally representative NIH PROMIS metric and each study using Monte Carlo simulations. This analysis served to verify the physical functioning trait across datasets was not only on the same scale but also had the same metric. We simulated 100,000 observations based on empirically available polychoric correlations and item thresholds. Using this simulated dataset, we calculated factor scores from dataset-specific sets of items. We compared these study-specific factor scores to known true factor scores based on the full set of physical functioning questions using correlations and Bland-Altman plots [43]. Differences would imply that scores obtained from the smaller set of measures used in individual studies are biased representations of the true scores.

## Results

### Participants

The pooled sample had a mean age of 75.9 years (SD = 9.9 years) with an age range of 42.9 to 105.1 years (Table 1). The mean number of years in a study were 3.3 years (SD = 2.0 years). The pooled sample was mostly female (75.7%) and nonwhite (53.0% black, 9.0% Hispanic, 34.9% non-Hispanic white, 3.1% other), indicating representation of multiple races/ethnicities across studies. Approximately 23.3% (N = 561) were high school graduates, 45.5% (N = 1,095) had some college, 14.0% (N = 337) held a college degree, and 9.8% (N = 237) held a post-graduate degree. BECT contributed the most participants (N = 702) in the pooled sample, followed by the REACH II (N = 670) and ABLE (N = 319) studies (Table 1).

**Table 1:** Characteristics of study sample (N = 2,556).

Characteristic	Overall	Advancing Better Living for Elders (ABLE)	Advancing Caregiver Training (ACT)	Alzheimer's Quality of Life (ALZQOL)	Baltimore Experience Corps Trial (BECT)	Beat the Blues (BTB)	Care of Persons with Dementia in their Environments (COPE)	Resources for Enhancing Alzheimer's Caregiver Health (REACH II)	Tailored Activity Program (TAP)
Sample size	N = 2,556	N = 319	N = 272	N = 88	N = 702	N = 208	N = 237	N = 670	N = 60
Age (in years), mean (SD)	75.9 (9.9)	79.0 (5.9)	82.4 (8.4)	81.7 (8.0)	67.4 (5.9)	69.6 (8.7)	82.7 (8.7)	79.0 (9.2)	79.4 (9.4)
Female, n (%)	1,936 (75.7)	261 (81.8)	220 (80.9)	78 (88.6)	596 (84.9)	163 (78.4)	202 (85.2)	390 (58.2)	26 (43.3)
Race/Ethnicity, n (%)									
White	893 (34.9)	168 (52.7)	191 (70.2)	67 (76.1)	34 (4.8)	0 (0.0)	167 (70.5)	220 (32.8)	46 (76.7)
African American	1,354 (53.0)	145 (45.5)	73 (26.8)	19 (21.6)	626 (89.2)	208 (100.0)	62 (26.2)	209 (31.2)	12 (20.0)
Hispanic	231 (9.0)	2 (0.6)	5 (1.8)	2 (2.3)	9 (1.3)	0 (0.0)	4 (1.7)	207 (30.9)	2 (3.3)
Other	78 (3.1)	4 (1.3)	3 (1.1)	0 (0.0)	33 (4.7)	0 (0.0)	4 (1.7)	34 (5.1)	0 (0.0)
Education, n (%)									
Less than high school	177 (7.4)	0 (0.0)	25 (9.2)	2 (2.3)	94 (13.4)	44 (21.2)	7 (3.0)	5 (1.0)	0 (0.0)
High school graduate	561 (23.3)	0 (0.0)	69 (25.4)	21 (23.9)	182 (25.9)	61 (29.3)	66 (27.9)	146 (28.0)	16 (26.7)
Some college	1,095 (45.5)	295 (92.5)	83 (30.5)	33 (37.5)	235 (33.5)	68 (32.7)	74 (31.2)	292 (55.9)	15 (25.0)
College degree	337 (14.0)	20 (6.3)	62 (22.8)	10 (11.4)	79 (11.3)	35 (16.8)	46 (19.4)	67 (12.8)	18 (30.0)
Post-graduate degree	237 (9.8)	4 (1.3)	33 (12.1)	22 (25.0)	112 (16.0)	0 (0.0)	44 (18.6)	12 (2.3)	10 (16.7)
Years in study, mean (SD)	3.3 (2.0)	2 (0.8)	2 (0.8)	1 (0.0)	5.8 (1.9)	2 (0.8)	2 (0.0)	1.7 (0.4)	3 (0.0)
Number of study visits, n (%)									
1	320 (12.5)	0 (0.0)	0 (0.0)	88 (100.0)	60 (8.6)	0 (0.0)	0 (0.0)	172 (25.7)	0 (0.0)
2	752 (29.4)	0 (0.0)	0 (0.0)	0 (0.0)	17 (2.4)	0 (0.0)	237 (100.0)	498 (74.3)	0 (0.0)
3	884 (34.6)	319 (100.0)	272 (100.0)	0 (0.0)	25 (3.6)	208 (100.0)	0 (0.0)	0 (0.0)	60 (100.0)
4	26 (1.0)	0 (0.0)	0 (0.0)	0 (0.0)	26 (3.7)	0 (0.0)	0 (0.0)	0 (0.0)	0 (0.0)
5	46 (1.8)	0 (0.0)	0 (0.0)	0 (0.0)	46 (6.6)	0 (0.0)	0 (0.0)	0 (0.0)	0 (0.0)
6	106 (4.2)	0 (0.0)	0 (0.0)	0 (0.0)	106 (15.1)	0 (0.0)	0 (0.0)	0 (0.0)	0 (0.0)
7	422 (16.5)	0 (0.0)	0 (0.0)	0 (0.0)	422 (60.1)	0 (0.0)	0 (0.0)	0 (0.0)	0 (0.0)
Cronbach's α for physical functioning items	0.89	0.84	0.92	0.90	0.92	0.84	0.92	0.96	0.89

SD = standard deviation

### Indicators of physical functioning

**Fig 1:**
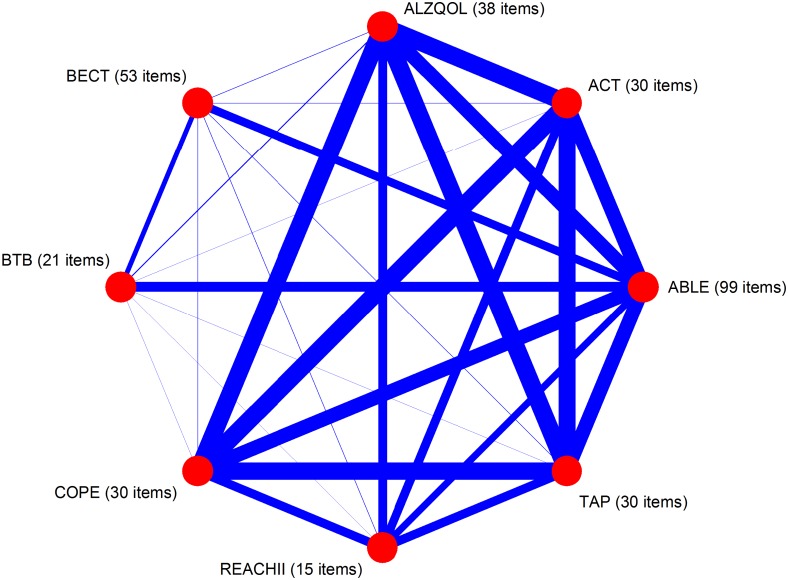
Network plot of physical functioning items. Advancing Better Living for Elders (ABL), Advancing Caregiver Training (ACT), Alzheimer’s Quality of Life (ALZ), Beat the Blues (BTB), Care of Persons with Dementia in their Environments (COP), Resources for Enhancing Alzheimer’s Caregiver Health II (REACH II), Tailored Activity Program (TAP), and Baltimore Experience Corps Trial (BECT).

There were 21 physical functioning items in BTB, 99 items in ABLE, 53 items in BECT, 38 items in ALZQOL, 30 items in ACT, 30 items in COPE, 30 items in TAP, and 15 items in REACH II. Cronbach’s α in each dataset were each above 0.80 (Table 1). In the network plot in Fig 1, line thicknesses linking each study are proportional to the number of physical functioning items in common between each dataset. Datasets of community-living older adults with dementia (ACT, ALZQOL, COPE, REACH II, TAP) tended to have more items in common with each other than with datasets of participants without dementia (BECT, BTB).

### Statistical analysis

#### Estimation of the physical functioning factor

**Fig 2:**
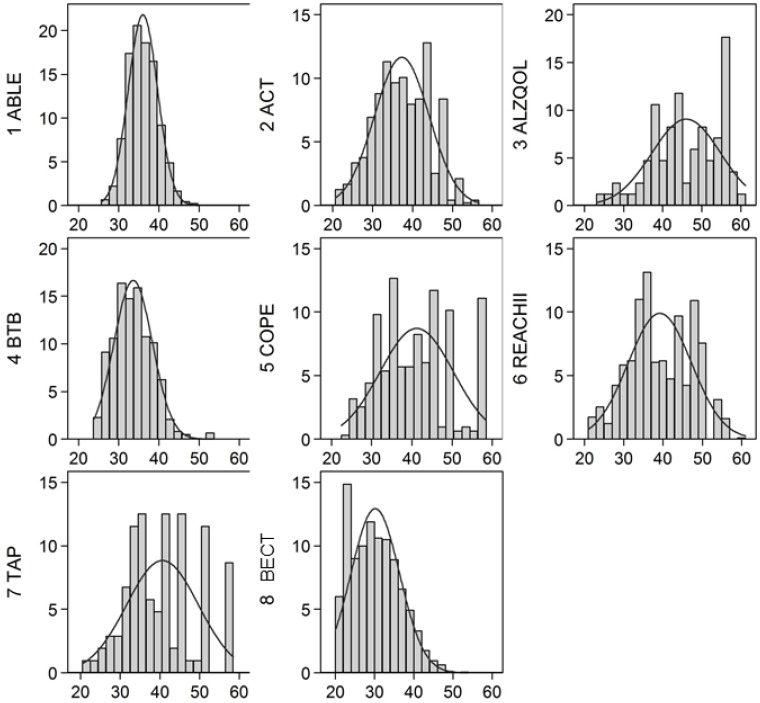
Distribution of externally scaled physical functioning factor by dataset. The x-axis is the range of the summary physical functioning score. The y-axis shows the proportion of participants in each panel who have a physical functioning score specified on the x-axis. Advancing Better Living for Elders (ABL), Advancing Caregiver Training (ACT), Alzheimer’s Quality of Life (ALZ), Beat the Blues (BTB), Care of Persons with Dementia in their Environments (COP), Resources for Enhancing Alzheimer’s Caregiver Health II (REACH II), Tailored Activity Program (TAP), and Baltimore Experience Corps Trial (BECT).

Factor loadings and thresholds estimated by the model are in S1 Table, and show a wide distribution across the range of the physical functioning latent variable. The distribution of the summary physical functioning factor was approximately normally distributed in each study, with ceiling effects for ACT, COPE, and TAP (Fig 2). High values on the physical functioning measure indicate more disability.

Items in S1 Table are sorted by the first threshold. Lower item thresholds indicate less disability or better physical functioning. Many physical functioning items with the lowest thresholds reflect IADLs, such as difficulty taking medication (threshold = -6.97), while many items with higher thresholds are ADLs (e.g., transferring in and out of bed, threshold = 1.17).

#### Precision

**Fig 3:**
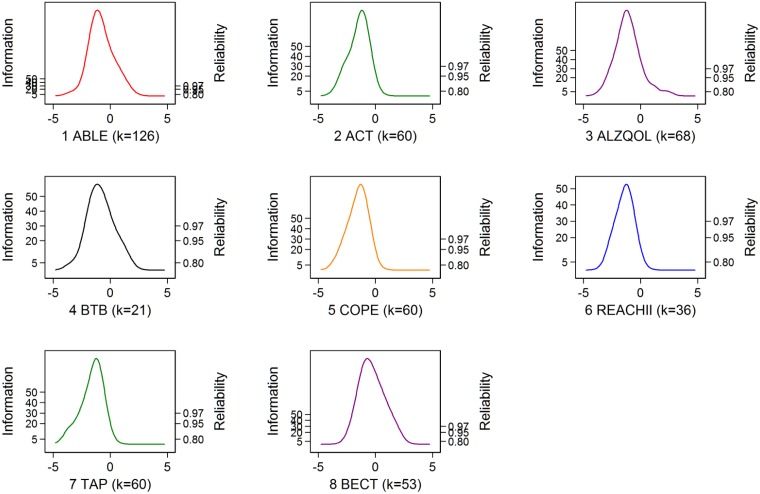
Precision of the summary physical functioning factor. Precision of the physical function score over the range of physical functioning. The information of the physical functioning is plotted over the range of functional ability for the summary factor score derived from using ADL/IADL items for each of the eight datasets. Reliability is excellent across the observed score range in each dataset with the lowest reliability being associated with the REA dataset. Reliability = I–I/Information = I–(standard error of measurement)^2^. Advancing Better Living for Elders (ABL), Advancing Caregiver Training (ACT), Alzheimer’s Quality of Life (ALZ), Beat the Blues (BTB), Care of Persons with Dementia in their Environments (COP), Resources for Enhancing Alzheimer’s Caregiver Health II (REACH II), Tailored Activity Program (TAP), and Baltimore Experience Corps Trial (BECT).

The precision of the measurement model of the physical functioning factors was evaluated by test information plots (Fig 3). The lowest precision in each dataset, at the extremes of the distribution, was 0.80. Because precision is a function of the number and quality of items, not sample size, ABLE contributed the most items to the summary physical functioning factor (99 items) and thus offered the most precise estimation of physical functioning, followed by BECT (53 items), ALZQOL (38 items), ACT (30 items), COPE (30 items), TAP (30 items), BTB (21 items), and REACH II (15 items) (Fig 3).

In BECT, we compared the summary physical functioning factor to an average of z-scored items (Table 2). Both scores had similar rates of worsening of physical functioning (0.431 standard deviation (SD) units/year for the factor versus 0.452 SD units/year for the z-score average). The model-estimated standard deviation was larger using the z-scored average of items (SD = 0.382) than the factor (SD = 0.257). Thus, the sample size required to have 80% power to detect 25% decline in physical functioning was 50% smaller for the summary factor (N = 558) compared to the z-score (N = 1,120).

**Table 2:** Precision in estimation of rate of change in physical functioning: Results from BECT (N = 702).

	Mean	Standard Deviation	Sample Size necessary to detect 25% change with 80% power
Summary Factor	0.431	0.257	558
Z-Scored Average of Items	0.452	0.382	1120

Estimated means and standard errors were calculated from random effects models of each physical functioning predictor regressed on time since start of study. Each score was standardized to have a mean of 50 and standard deviation of 10.

BECT = Baltimore Experience Corps Trial

#### Validity

**Fig 4:**
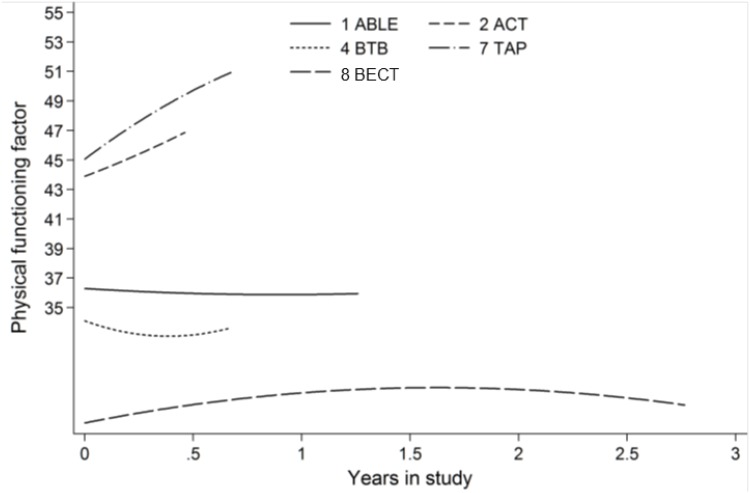
Face validity using model-estimated change in functioning over time since study entry. A higher physical functioning factor represented greater functional limitation. Participants of ABL, BTB, and BECT were at risk for developing dementia, so baseline physical functioning factor was lower in these studies than in TAP and ACT, which consisted of participants with a diagnosis of dementia. The model-estimated change in functioning decreased over time in TAP and ACT, which the model-estimate change slightly decreased or remained constant in BTB and ABL. The BECT had the longest period of follow-up and the lowest physical functioning factor across time since study entry, as compared with the other studies. There is a slight increase from study entry to 1.5 years and a slight decrease from 1.5 years to 2.75 years since study entry. Advancing Better Living for Elders (ABL), Advancing Caregiver Training (ACT), Beat the Blues (BTB), Tailored Activity Program (TAP), and Baltimore Experience Corps Trial (BECT).

We compared average rates of change in the physical functioning factor using data from studies with more than two visits (ACT, ABLE, BTB, TAP, and BECT). We hypothesized that samples with more impaired participants in institutionalized settings have a worse (higher) physical functioning factor score and deteriorate more steeply over time. Accordingly, in Fig 4, average trajectories for ABLE, BTB, and BECT were less impaired than in ACT and TAP and showed relatively minimal change. Participants in ACT and TAP on average had worse and worsening physical functioning (Fig 4).

Mean levels of physical functioning are provided in Table 3 by category of objective functioning measures in ALZQOL and BECT. Better mobility measured by the Timed Up and Go Task in ALZQOL was associated with less disability according to the estimated physical functioning factor in a dose-response fashion. Similarly, successful tandem balance, faster gait, faster time to complete chair stands, and stronger grip strength were each associated with less impaired physical functioning scores in BECT (Table 3). Since there were few frail participants (N = 3), we combined pre-frail and frail participants as one group. Higher mean physical functioning score was associated with pre-frail/frail status (Table 3). S2 Table provides mean levels of physical functioning by sex and age strata to evaluate criterion validity across these subgroups.

**Table 3:** Convergent criterion validity of the physical functioning measure.

Objective Functioning Measures	N	Mean physical functioning score	p-value relative to reference
Timed Up and Go Task (ALZQOL)			
Normal mobility (<20 s)	58	44.9	REF
Problems (21–30 s)	21	53.0	<0.01
Tandem balance (BECT)			
Held 10+ s	479	25.4	REF
Not held	164	26.6	0.01
Gait speed (BECT)			
Faster than 0.8 m/s	342	25.3	REF
Slower than 0.8 m/s	346	27.6	<0.01
Chair stands, time to complete (BECT)			
Faster than median (12.6 s)	312	24.5	REF
Slower than median	313	26.3	<0.01
Grip strength (BECT)			
Stronger than median (26 kg)	332	25.1	REF
Weaker than median	326	28.1	<0.01
Physical Frailty* (BECT)			
Robust	483	24.9	REF
Pre-frail/Frail	219	27.1	<0.01

Estimated means are from linear regression adjusted by age and sex. ALZQOL: Alzheimer’s Quality of Life; BECT: Baltimore Experience Corps Trial

*Physical frailty was defined by Fried’s criteria: weight loss, exhaustion, physical activity, walk time, grip strength; Robust: 0 criteria met, Pre-frail: 1–2 criteria met, Frail: 3+ criteria met

#### Simulation

To evaluate the quality of the link between the nationally representative NIH PROMIS metric and each study, we compared study-specific factor scores in simulated data to known true factor scores based on the full set of physical functioning questions using correlations and Bland-Altman plots (S1 Fig). The plots revealed minimal evidence of systematic bias along the range of physical functioning, in the sense that bias is the mean difference between a true value and an observed value. At higher (more impaired) levels of physical functioning the scores were notably less precise in ACT, COPE, REACH II, and TAP.

## Discussion

The purpose of this work was to derive and validate a factor for physical functioning based on 232 items from traditional physical functioning scales administered in eight existing datasets. This psychometrically derived factor balances precision over a broad range with content and criterion validity. The standardized physical functioning metric is scaled to the NIH PROMIS metric, is internally consistent, is precise across a broad range of low and average levels of physical functioning, and produced more precise estimates of change than an alternative approach to summarizing physical functioning. The factor demonstrated criterion validity with respect to objective measures of physical functioning.

This study addresses the challenge of comparing findings across studies using different but overlapping measures of physical functioning. Data provided in S1 Table could be used to select a reduced set of items based on physical functioning levels of people being assessed in future studies. We externally scaled the factor to NIH PROMIS normative data so that raw scores are interpretable, however this step is unnecessary in studies in which national representativeness is unnecessary.

Our study has three primary advantages. First, estimates based on this measure are scaled to NIH PROMIS normative data, and can thus serve as a common tool to directly compare physical functioning to existing studies with findings from new studies. Second, larger sample sizes in a pooled sample enable an array of novel scientific questions to be addressed. Namely, questions involving effect modification that require subgrouping, or when an outcome is particularly rare, are made possible by combining samples and maximizing summary measures’ comparability, precision, and classification quality. Third, we used eight randomized trials which included a wide range of types and levels of physical impairment. Thus, we show that harmonization works with disparate datasets and yields a valid, reliable, and usable physical function factor. Our approach demonstrates that existing datasets can be mined similarly for the purpose of deriving measures and thus maximizing efficiencies in cross-study comparisons.

Besides strengths, several limitations of the study must be highlighted. First, the dimensionality of the NIH PROMIS physical functioning item bank is based on quantitative and qualitative criteria, neither of which are infallible. Since physical functional ability integrates several components, physical functioning might be subdivided into subdomains such as muscle strength, coordination, cognition, and social and environmental context (e.g., [44]). Relatedly, some physical functioning indicators may measure physical functioning differently in other samples. We addressed this limitation by testing and adjusting for differential item functioning by dataset. A third limitation is that the quality of a score is contingent upon the number and psychometric characteristics of questions gathered in a study; poor or insensitive measurements will not produce a good summary measure regardless of the scoring method. Along the same reasoning, self-reported questionnaires about IADL and ADL functioning can be less reliable than direct measurements of fitness or activity levels [45]. Fourth, in presenting data on criterion validity our goal was to demonstrate external validity of the physical functioning construct. While the factor score corresponds with available physical functioning tests in available studies, replication in other data sources is necessary to further demonstrate generalizability to other populations. Relatedly, we used measures for criterion validity that we had available; this work can be extended to other data sources—old and new—that have available data on, for example, aerobic capacity and flexibility. A final study limitation is that the harmonization procedure we implemented relies on the availability of common items or questions across studies; studies without overlapping items cannot be harmonized. Relatedly, linking to the NIH PROMIS metric is contingent on the availability of items overlapping with the NIH PROMIS item bank.

## Conclusions

We repurposed widely used ADL and IADL indicators from eight existing datasets and publicly available data from the NIH PROMIS initiative to derive, externally scale, and validate a summary measure of physical functioning. The measure was internally consistent, reliable especially over average-to-low levels of physical functioning, and demonstrated better power to detect differences in change than other commonly used methods to derive measures. Our measure had concurrent criterion validity with respect to objective measures of physical functioning. The harmonization is extensible, provided a study has items overlapping with the NIH PROMIS item bank, and can be used to integrate findings across existing and future research on physical functioning.

## Supporting information

S1 Fig Bland-Altman plots of dataset-specific physical functioning factor vs the overall physical functioning factor. Bland-Altman plots graph the difference in 2 scores on the Y axis against their mean on the X axis, and tell us about bias across the range of scores. The reference in all these plots is the true simulated theta score. The vertical spread tells us about precision of the scores. The scores are on a T-score metric (m50, sd10), so a 2 point difference corresponds to an imprecision of 0.2 SD. Advancing Better Living for Elders (ABL), Advancing Caregiver Training (ACT), Alzheimer’s Quality of Life (ALZ), Beat the Blues (BTB), Care of Persons with Dementia in their Environments (COP), Resources for Enhancing Alzheimer’s Caregiver Health II (REA), Tailored Activity Program (TAP), and Baltimore Experience Corps Trial (BEC).
(PDF)

S1 Table Physical functioning items and their model-estimated thresholds from the IRT model. (DOCX)

S2 Table Convergent criterion validity of the physical functioning measure by sex and age. * We used an age cutoff of 65 years in the BECT study but 80 in the ALZQOL study because of too few participants (N = 3) younger than 65 years in that study. ** Physical frailty was defined by Fried’s criteria: weight loss, exhaustion, physical activity, walk time, grip strength; Robust: 0 criteria met, Pre-frail: 1–2 criteria met, Frail: 3+ criteria met.
(DOCX)
