# Supplementary material for: E pluribus unum: Harmonization of physical functioning across intervention studies of middle-aged and older adults
Source: PLoS One. 2017 Jul 28;12(7):e0181746. doi: 10.1371/journal.pone.0181746 (PMC5533461; doi:10.1371/journal.pone.0181746)
Supplement: S1 Fig — Bland-Altman plots graph the difference in 2 scores on the Y axis against their mean on the X axis, and tell us about bias across the range of scores. The reference in all these plots is the true simulated theta score. The vertical spread tells us about precision of the scores. The scores are on a T-score metric (m50, sd10), so a 2 point difference corresponds to an imprecision of 0.2 SD. Advancing Better Living for Elders (ABL), Advancing Caregiver Training (ACT), Alzheimer’s Quality of Life (ALZ), Beat the Blues (BTB), Care of Persons with Dementia in their Environments (COP), Resources for Enhancing Alzheimer’s Caregiver Health II (REA), Tailored Activity Program (TAP), and Baltimore Experience Corps Trial (BEC). (PDF) [file pone.0181746.s001.pdf]

**S3 Figure. Bland-Altman plots of dataset-specific physical functioning factor vs the overall physical functioning factor.**

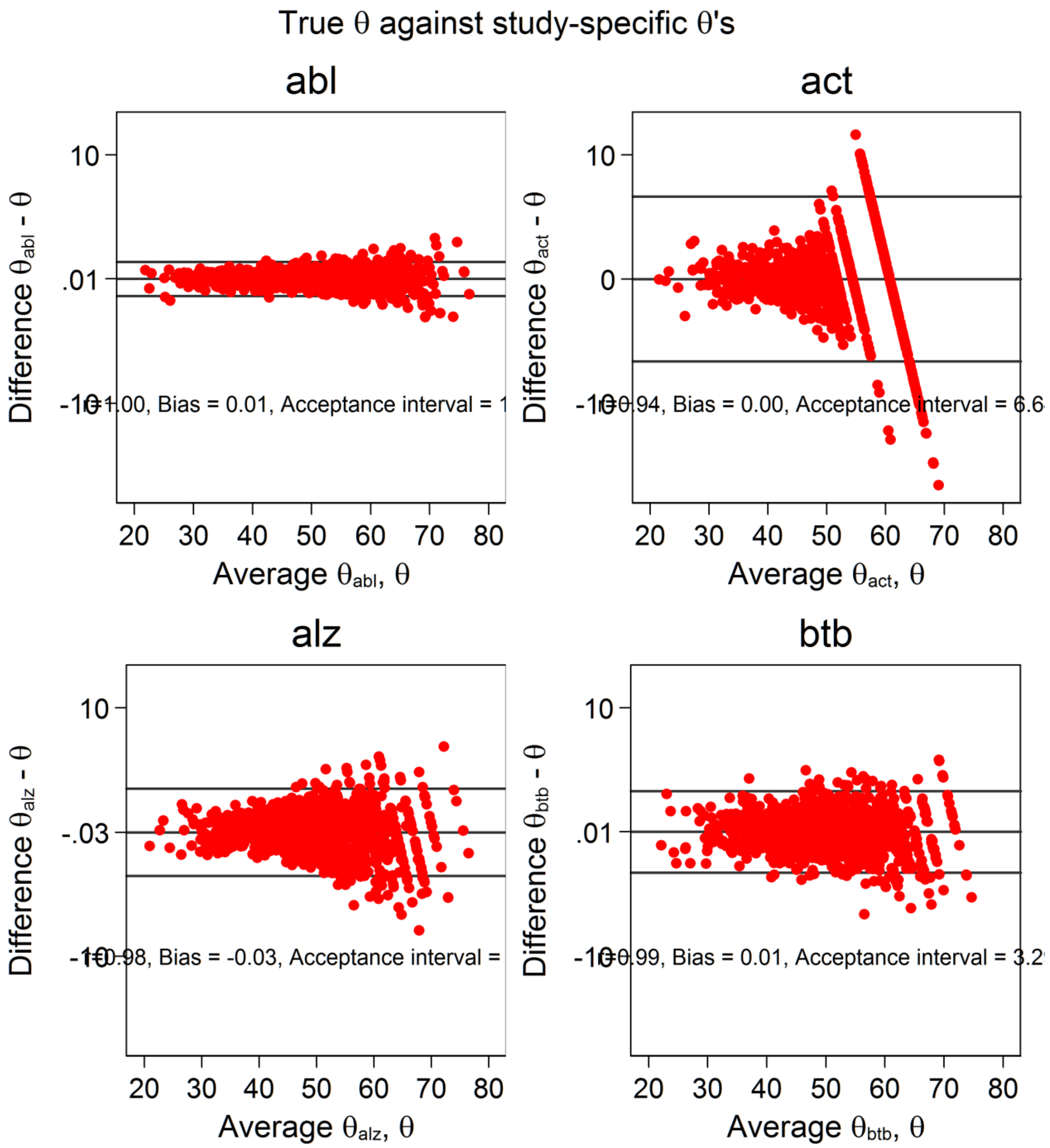

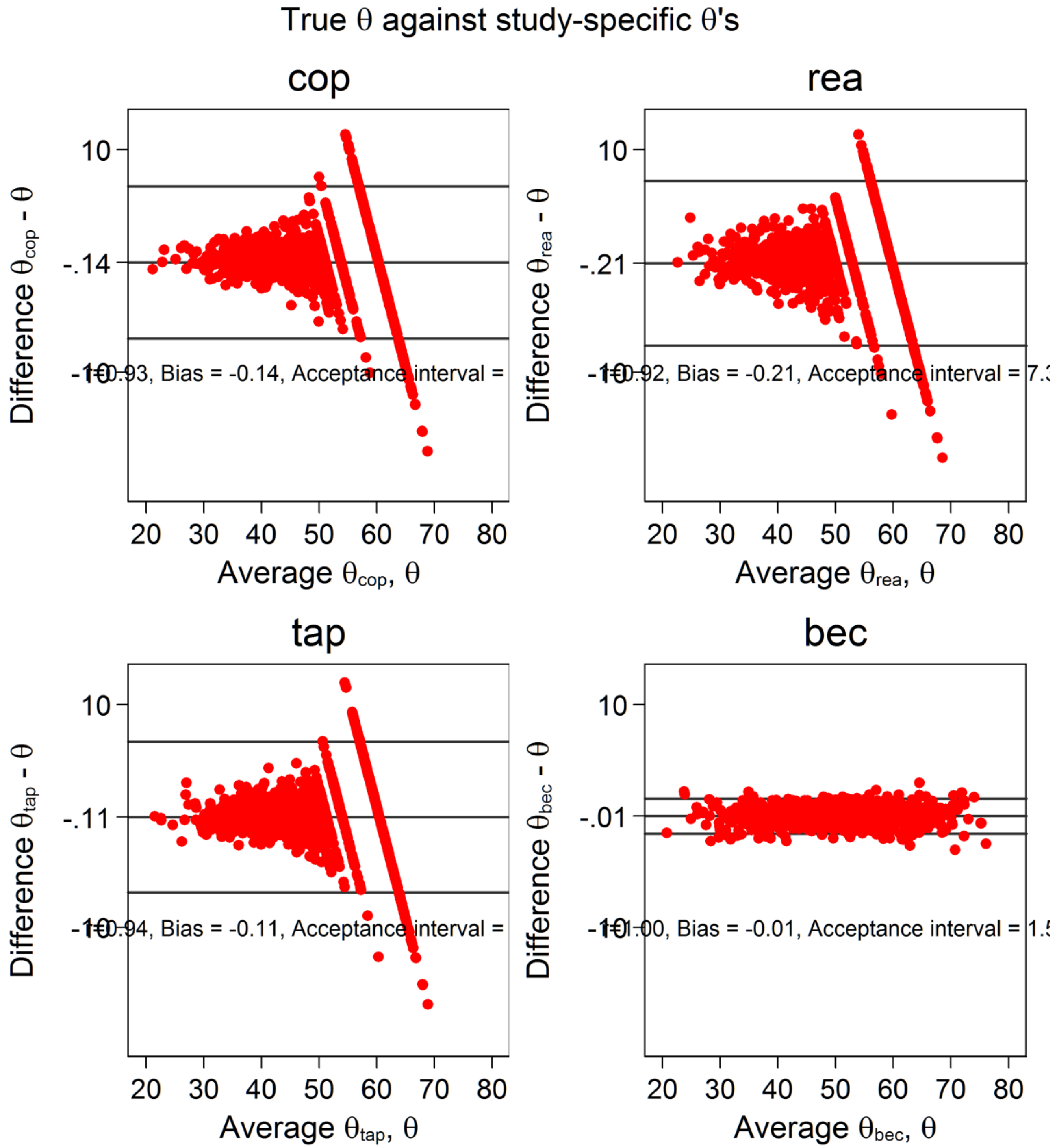


Bland-Altman plots graph the difference in 2 scores on the Y axis against their mean on the X axis, and tell us about bias across the range of scores. The reference in all these plots is the true simulated theta score. The vertical spread tells us about precision of the scores. The scores are on a T-score metric (m50, sd10), so a 2 point difference corresponds to an imprecision of 0.2 SD.

Advancing Better Living for Elders (ABL), Advancing Caregiver Training (ACT), Alzheimer's Quality of Life (ALZ), Beat the Blues (BTB), Care of Persons with Dementia in their Environments (COP), Resources for Enhancing Alzheimer's Caregiver Health II (REA), Tailored Activity Program (TAP), and Baltimore Experience Corps Trial (BEC)
