# Supplementary material for: E pluribus unum: Harmonization of physical functioning across intervention studies of middle-aged and older adults
Source: PLoS One. 2017 Jul 28;12(7):e0181746. doi: 10.1371/journal.pone.0181746 (PMC5533461; doi:10.1371/journal.pone.0181746)
Supplement: S1 Table — (DOCX) [file pone.0181746.s002.docx]

Supplemental table 1. Physical functioning items and their model-estimated thresholds from the IRT model

| **Physical functioning item** | **Raw threshold** |
| --- | --- |
| In the past month, how difficult was it for you to take medication? | -6.97, -0.88, 0.11, 1.58, 3.50 |
| ABLE: Do you get help from another person to feed yourself? | -6.89 |
| Does your (CR) need an assistive device (for example a magnifying glass, special calculator) for handling his/her finances, does he/she take more than reasonable time for handling his/her finances, or is there a concern for safety? | -6.73 |
| COPE: Do you use any kind of assistive device or special equipment (for example a pill dispenser or crusher) to help you take medication? | -6.60 |
| COPE: Do you get help from another person to take medication? | -6.23 |
| ABLE: Do you use any kind of assistive device or special equipment (for example a rocker knife, dycem, or any adapted utensils) to help you feed yourself? | -6.18 |
| TAP: Do you use any kind of assistive device or special equipment (for example a pill dispenser or crusher) to help you take medication? | -5.86 |
| ACT: During the past week has your (CR) needed any kind of help with doing laundry? | -5.85 |
| TAP: Do you get help from another person to take medication? | -5.85 |
| ABLE: Do you get help from another person to use the toilet, including getting on and off the toilet? | -5.84 |
| ALZQOL: During the past four weeks, how much of the time has your physical health or emotional problems interfered with your social activities like visiting with friends, relatives, etc.? | -5.63, -4.18, -2.95, -0.49 |
| REACH II: During the past week has your (CR) needed any kind of help with doing laundry? | -5.59 |
| Do you get help from another person to take medication? | -5.54 |
| Do you get help from another person to prepare your own meals? | -5.46 |
| Do you restrict or limit the way you do activities to make it easier, such as carrying fewer items or not doing as much at one time? | -5.42 |
| ALZQOL: Does your (CR) need an assistive device (for example a cane or walker, a cart to transport item) for doing laundry, does he/she take more than reasonable time for doing laundry, or is there a concern for safety? | -5.36 |
| During the past week has your (CR) needed any kind of help handling his/her finances? | -5.35 |
| ABLE: Do you get help from another person to take medication? | -5.34 |
| REACH II: Do you get help from another person to take medication? | -5.08 |
| How much physical pain have you had during the past four weeks? | -5.01, -4.07, -3.31, -1.74, 0.36 |
| ALZQOL: Does your (CR) need an assistive device (for example a cane or walker, a cart to transport item) for doing laundry, does he/she take more than reasonable time for doing laundry, or is there a concern for safety? | -4.99 |
| REACH II: During the past week has your (CR) needed any kind of help with traveling by car, bus, etc.? | -4.91 |
| ABLE: Do you get help from another person to prepare your own meals? | -4.78 |
| COPE: During the past week has your (CR) needed any kind of help with traveling by car, bus, etc.? | -4.66 |
| COPE: Does your (CR) need an assistive device (for example seating adaptation) for traveling by car, bus, etc., does he/she take more than reasonable time for traveling by car, bus, etc., or is there a concern for safety? | -4.66 |
| ACT: Does your (CR) need an assistive device (for example seating adaptation) for traveling by car, bus, etc., does he/she take more than reasonable time for traveling by car, bus, etc., or is there a concern for safety? | -4.63 |
| getting up from the floor | -4.52, -2.56, -1.35, 0.40 |
| In the past month, how difficult was it for you to walk indoors, such as around your home? | -4.49, -3.84, -2.75, -1.72 |
| Do you take more time to do an activity, pace yourself, or take rest breaks? | -4.43 |
| REACH II: Do you get help from another person to do errands, such as grocery shopping or shopping for personal items? | -4.37 |
| How much difficulty have you had falling asleep or staying asleep, during the past four weeks? | -4.29, -3.24, -2.42, -0.23 |
| BECT: Do you get help from another person to bathe or shower? | -4.27 |
| ACT: During the past week has your (CR) needed any kind of help with doing laundry? | -4.26 |
| Could you walk up and down every aisle in a grocery store without sitting down to rest or leaning on a cart? | -4.08 |
| ACT: Do you get help from another person to use the telephone? | -4.05 |
| REACH II: Do you get help from another person to do errands, such as grocery shopping or shopping for personal items? | -4.05 |
| Using public transportation? | -3.99, -3.13, -2.69, -2.35 |
| REACH II: During the past week has your (CR) needed any kind of help with doing laundry? | -3.97 |
| ALZQOL: During the past four weeks, how much did pain interfere with your normal work, including both work outside the home and housework? Did it interfere? | -3.92, -2.99, -2.12, -0.12 |
| COPE: During the past week has your (CR) needed any kind of help with traveling by car, bus, etc.? | -3.90 |
| rip open a package of snack food using only your hands | -3.87, -2.88, -2.20, -1.38 |
| REACH II: Do you get help from another person to do light housework? | -3.83 |
| ACT: Do you use any kind of assistive device or special equipment (for example a tub bench, handheld shower, or grab bars) to help you bathe or shower? | -3.82 |
| COPE: Does your (CR) need an assistive device (for example seating adaptation) for traveling by car, bus, etc., does he/she take more than reasonable time for traveling by car, bus, etc., or is there a concern for safety? | -3.81 |
| ALZQOL: Do you use any kind of assistive device or special equipment (for example a tub bench, handheld shower, or grab bars) to help you bathe or shower? | -3.80 |
| Taking care of a family member? | -3.79, -3.36, -3.15, -2.91 |
| first, consider moderate activities, such as moving a table, pushing a vacuum cleaner, bowling, or playing golf. Does your health now limit you a lot, limit you a little, or not limit you at all? | -3.78, -3.32, -2.86, -2.35 |
| Raising your arms above your head (to comb your hair or put away groceries)? | -3.77, -3.08, -2.39, -1.67 |
| REACH II: Do you get help from another person to do light housework? | -3.76 |
| Do you use a different way of moving your body for any of your activities (such as the way that you get in and out of a bed, chair, or car)? | -3.75 |
| going up and down 3 flights of stairs inside, using a handrail | -3.75, -2.44, -0.99, 0.64 |
| Do you put items together or within reach to make it easier to get things without bending or reaching up high? | -3.71 |
| ACT: Do you use any kind of assistive device or special equipment (for example a microwave, adapted utensils, dycem, reacher) to help you prepare your own meals? | -3.69 |
| In the past month, how difficult was it for you to get in and out of a car? | -3.57, -2.68, -1.94, -1.07, 1.88 |
| Participating in leisure activities or hobbies (such as gardening, playing cards, handiwork, woodworking, etc.)? | -3.56, -3.06, -2.41, -1.73 |
| In the past month, how difficult was it for you to move in and out of bed? | -3.53, -2.72, -2.07, -1.18, 2.09 |
| ACT: Do you use any kind of assistive device or special equipment (for example a rocker knife, dycem, or any adapted utensils) to help you feed yourself? | -3.52 |
| Do you sit instead of stand while doing an activity? | -3.50 |
| pouring from a large pitcher | -3.46, -2.94, -2.43 |
| In the past month, how difficult was it for you to dress above the waist? | -3.41, -2.99, -2.42, -1.72 |
| REACH II: During the past week has your (CR) needed any kind of help with traveling by car, bus, etc.? | -3.38 |
| Opening containers or removing wrappers (lids from unopened jars, snack foods, milk cartons)? | -3.38, -2.91, -2.30, -1.67 |
| In the past month, how difficult was it for you to move in and out of a chair? | -3.37, -2.86, -2.28, -1.55, 2.68 |
| Do you give up or avoid an activity that you think might be too difficult, such as climbing stairs? | -3.36 |
| In the past month, how difficult was it for you to bathe or shower? | -3.35, -2.65, -2.07, -1.48, 1.28 |
| In the past month, how difficult was it for you to feed yourself? | -3.34, -2.87, -2.50 |
| Do you use any kind of assistive device or special equipment (for example a cart to transport items) to help you do errands, such as grocery shopping or shopping for personal items? | -3.31 |
| ACT: Do you use any kind of assistive device or special equipment (for example a tub bench, handheld shower, or grab bars) to help you bathe or shower? | -3.30 |
| COPE: Do you use any kind of assistive device or special equipment (for example a rocker knife, dycem, or any adapted utensils) to help you feed yourself? | -3.25 |
| ALZQOL: Do you get help from another person to use the telephone? | -3.22 |
| How confident are you that you can find a way to get light housework done? | -3.21, -2.27 -1.00 0.45 |
| Do you hold onto or lean on things while moving or walking around your home? | -3.19 |
| Gripping with your hands, such as a cup, faucet, or can? | -3.19, -2.51, -1.94, -1.18 |
| opening a heavy, outside door | -3.19, -1.06, 0.60, 4.15 |
| In the past month, how difficult was it for you to dress from the waist down? | -3.18, -2.73, -2.08, -1.35 |
| Do you use clothing or shoes that are easier to put on, take off or walk around in? | -3.15 |
| Do you use special items to help make it easier for you to do activities (such as light-handled items, microwave, etc.)? | -3.12 |
| COPE: Do you use any kind of assistive device or special equipment (for example a reacher, button hooks, velcro clothing) to help you dress above the waist? | -3.12 |
| In the past month, how difficult was it for you to use the toilet, including getting on and off the toilet? | -3.11, -2.78, -2.21, -1.46 |
| How confident are you that you can find a way to climb one flight of stairs? | -3.09, -2.08, -0.46, 1.30 |
| Driving a car? | -3.09, -2.80, -2.72, -2.71 |
| taking part in active recreation | -3.04, -2.09, -0.92, 0.66 |
| Do you plan ahead to save your energy (such as preparing more food at one time and freezing or storing leftovers, calling ahead to order groceries, etc.)? | -3.03 |
| ACT: Do you get help from another person to prepare your own meals? | -2.99 |
| In the past month, how difficult was it for you to prepare your own meals? | -2.96, -2.52, -2.08, -1.55, -0.58 |
| How confident are you that you can find a way to walk one block? | -2.96, -2.04, -0.67, 1.06 |
| REACH II: Do you get help from another person to use the toilet, including getting on and off the toilet? | -2.94 |
| Bending or stooping? | -2.92, -2.21, -1.63, -0.90 |
| washing dishes, pots, and utensils by hand while standing at a sink | -2.91, -2.54, -2.08, -1.56 |
| ABLE: Do you get help from another person to use the telephone? | -2.90 |
| TAP: Do you get help from another person to take medication? | -2.90 |
| ALZQOL: Do you use any kind of assistive device or special equipment (for example a rocker knife, dycem, or any adapted utensils) to help you feed yourself? | -2.88 |
| COPE: Do you get help from another person to dress above the waist? | -2.87 |
| Do you get help from another person to use the toilet, including getting on and off the toilet? | -2.80 |
| taking part in regular fitness programs | -2.80, -1.73, -0.45, 1.21 |
| COPE: Do you get help from another person to take medication? | -2.78 |
| How confident are you that you can find a way to get errands done? | -2.76, -1.90 -0.49 0.89 |
| COPE: Do you get help from another person to use the toilet, including getting on and off the toilet? | -2.72 |
| COPE: Do you get help from another person to dress from the waist down? | -2.71 |
| Do you use any kind of assistive device or special equipment (for example a cane or walker, a cart to transport items, or large handled items) to help you do light housework? | -2.70 |
| sitting down in and standing up from a low, soft couch | -2.68, -1.79, -1.17, -0.46 |
| How confident are you that you can find a way to get in and out of a car? | -2.65, -1.66, -0.17, 1.57 |
| walking on a slippery surface outdoors | -2.65, -1.70, -0.94, 0.32 |
| traveling out of town for at least an overnight stay | -2.65, -1.76, -0.28, 1.20 |
| Lifting or carrying something as heavy as 10 pounds, such as a bag of groceries? | -2.63, -2.23, -1.79, -1.11 |
| Do you get help from another person to take medication? | -2.63 |
| ALZQOL: Do you get help from another person to use the toilet, including getting on and off the toilet? | -2.63 |
| TAP: Do you get help from another person to use the toilet, including getting on and off the toilet? | -2.63 |
| stepping on and off a bus | -2.61, -1.36, 0.08, 1.44 |
| ABLE: Do you use any kind of assistive device or special equipment (for example grab bars, commode, or a raised toilet seat) to help you use the toilet, including getting on and off the toilet? | -2.59 |
| REACH II: Do you get help from another person to feed yourself? | -2.56 |
| In the past month, how difficult was it for you to do errands, such as grocery shopping or shopping for personal items? | -2.55, -1.97, -1.45, -0.82, -0.21 |
| ALZQOL: Do you use any kind of assistive device or special equipment (for example a tub bench, handheld shower, or grab bars) to help you bathe or shower? | -2.54 |
| making a bed, including spreading and tucking in bedsheets | -2.53, -2.02, -1.52, -0.97 |
| How confident are you that you can find a way to move in and out of a chair? | -2.52, -1.42, 0.05, 2.42 |
| TAP: Do you use any kind of assistive device or special equipment (for example a pill dispenser or crusher) to help you take medication? | -2.51 |
| Do you get help from another person to get in and out of your own car? | -2.47 |
| picking up a kitchen chair and moving it, in order to clean | -2.45, -1.96, -1.49, -0.84 |
| ALZQOL: Do you get help from another person to feed yourself? | -2.44 |
| ALZQOL: Do you use any kind of assistive device or special equipment (for example a rocker knife, dycem, or any adapted utensils) to help you feed yourself? | -2.44 |
| providing care or assistance to others | -2.42, -1.22, 0.34, 1.94 |
| Do you use any kind of assistive device or special equipment (for example a cane, walker or wheelchair) to help you walk indoors, such as around your home? | -2.38 |
| Participating in community activities such as religious services, social activities or volunteer work? | -2.37, -1.67, -1.28, -1.13 |
| ALZQOL: Do you use any kind of assistive device or special equipment (for example grab bars, commode, or a raised toilet seat) to help you use the toilet, including getting on and off the toilet? | -2.37 |
| visiting friends and family in their homes | -2.36, -1.35, 0.79, 2.70 |
| In the past month, how difficult was it for you to walk one block? | -2.34, -1.84, -1.38, -0.90, -0.30 |
| In the past month, how difficult was it for you to do light housework? | -2.33, -1.81 -1.23 -0.49 0.35 |
| ACT: Do you use any kind of assistive device or special equipment (for example grab bars, commode, or a raised toilet seat) to help you use the toilet, including getting on and off the toilet? | -2.33 |
| COPE: Do you get help from another person to groom, such as brushing teeth, combing or brushing hair, washing hands, washing hands, washing face, and either shaving or applying makeup? | -2.33 |
| REACH II: Do you get help from another person to take medication? | -2.33 |
| taking care of the inside of your home (housemaking, laundry, etc.) | -2.32, -1.02, 1.09, 3.45 |
| How confident are you that you can find a way to bathe or shower? | -2.31, -1.36, 0.04, 1.47 |
| How confident are you that you can find a way to dress from the waist down? | -2.30, -1.39, 0.01, 2.07 |
| ABLE: Do you use any kind of assistive device or special equipment (for example large handled items, adapted faucets) to help you groom, such as brushing teeth, combing or brushing hair, washing hands, washing face, and either shaving or applying makeup? | -2.29 |
| BECT: Do you get help from another person to bathe or shower? | -2.24 |
| inviting people into your home for a meal or entertainment | -2.23, -1.26, 0.39, 2.13 |
| Lifting heavy objects? | -2.21, -1.74, -1.25, -0.66 |
| COPE: Do you use any kind of assistive device or special equipment (for example a pill dispenser or crusher) to help you take medication? | -2.21 |
| How confident are you that you can find a way to move in and out of bed? | -2.19, -1.23, 0.45, 2.25 |
| ACT: Do you get help from another person to feed yourself? | -2.18 |
| ACT: Do you use any kind of assistive device or special equipment (for example grab bars, commode, or a raised toilet seat) to help you use the toilet, including getting on and off the toilet? | -2.14 |
| ACT: Does your (CR) need an assistive device (for example seating adaptation) for TRAVELING BY CAR, BUS, ETC., does he/she take more than reasonable time for traveling by car, bus, etc., or is there a concern for safety? | -2.13 |
| running a short distance, such as to catch a bus | -2.12, -1.76, -1.24, -0.48 |
| How confident are you that you can find a way to dress above the waist? | -2.09, -1.11, 0.52, 2.45 |
| How confident are you that you can find a way to groom, such as brushing teeth, combing or brushing hair, washing hands, washing face, and either shaving or applying makeup? | -2.06, -1.05, 0.59, 2.65 |
| Do you do things to help your memory or make it easier to recognize objects (such as using labels or bright signs, etc.)? | -2.05 |
| Doing heavy work around the house like mowing the lawn, painting, or scrubbing? | -2.05, -1.49, -0.94, -0.22 |
| How confident are you that you can find a way to walk indoors, such as around your home? | -2.03, -1.03, 0.49, 2.65 |
| working at a volunteer job outside your home | -2.00, -0.92, 0.74, 1.93 |
| going out with others to public places such as restaurants | -2.00, -0.88, 0.91, 2.48 |
| taking part in organized, social activities | -2.00, -0.85, 0.89, 2.40 |
| COPE: Do you get help from another person to feed yourself? | -2.00 |
| Do you sponge bathe instead of using the shower or bathtub? | -1.99 |
| using a step stool to reach into a high cabinet | -1.99, -0.84, 0.04, 0.72 |
| reaching behind your back as if to put a belt through a belt loop | -1.97, -0.48, 1.09, 2.50 |
| stepping up and down from a curb | -1.96, -0.39, 1.16, 2.39 |
| COPE: Do you get help from another person to dress from the waist down? | -1.96 |
| COPE: Do you get help from another person to dress above the waist? | -1.96 |
| During the past four weeks, did you not do work or other regular activities as carefully as usual as a result of any emotional problems such as feeling depressed or anxious? | -1.92 |
| ALZQOL: Do you get help from another person to use the telephone? | -1.92 |
| ABLE: Do you use any kind of assistive device or special equipment (for example grab bars, commode, or a raised toilet seat) to help you use the toilet, including getting on and off the toilet? | -1.90 |
| In the past month, how difficult was it for you to climb one flight of stairs? | -1.89, -1.48, -1.05, -0.40, 0.79 |
| Do you use paid or unpaid services from an agency or person to help you with any of your activities? | -1.86 |
| ALZQOL: Do you get help from another person to feed yourself? | -1.86 |
| REACH II: Do you get help from another person to use the toilet, including getting on and off the toilet? | -1.85 |
| COPE: Do you get help from another person to groom, such as brushing teeth, combing or brushing hair, washing hands, washing hands, washing face, and either shaving or applying makeup? | -1.85 |
| carrying something in both arms while climbing a flight of stairs (eg laundry basket) | -1.84, -1.45, -1.03, -0.42 |
| Do you use any kind of assistive device or special equipment (for example a reacher, stocking donner) to help you dress from the waist down? | -1.81 |
| How confident are you that you can find a way to prepare your own meals? | -1.79, -0.96, 0.27, 1.55 |
| Overall, how satisfied have you been in the past month with the help that you have received from friends, neighbors, or family members for the activities that we just talked about? | -1.78, -0.48, 0.47 |
| reaching overhead while standing, as if to pull a light cord | -1.78, -1.54, -1.17, -0.54 |
| ACT: Do you use any kind of assistive device or special equipment (for example a microwave, adapted utensils, dycem, reacher) to help you prepare your own meals? | -1.77 |
| Managing your money, such as paying bills? | -1.72, -0.66, 0.09, 1.09, 2.57 |
| How confident are you that you can find a way to use the toilet, including getting on and off the toilet? | -1.66, -0.73, 0.85, 2.53 |
| taking care of household business and finances | -1.66, -0.61, 1.20, 2.96 |
| ABLE: Do you get help from another person to use the toilet, including getting on and off the toilet? | -1.63 |
| ABLE: Do you get help from another person to prepare your own meals? | -1.63 |
| Do you use any kind of assistive device or special equipment (for example a cane, walker or wheelchair) to help you walk one block? | -1.62 |
| Do you get help from another person to use the toilet, including getting on and off the toilet? | -1.60 |
| Do you use any kind of assistive device or special equipment (for example a stairglide, handrail, elevator) to help you climb one flight of stairs? | -1.59 |
| ACT: Do you get help from another person to use the telephone? | -1.56 |
| Climbing several flights of stairs? | -1.56, -0.97, -0.44, 0.25 |
| Do you use any kind of assistive device or special equipment (for example large numbers on the face or hearing augmentation) to help you use the telephone? | -1.55 |
| taking care of local errands | -1.55, -0.03, 2.11, 3.05 |
| During the past four weeks, have you accomplished less than you would like to as a result of any emotional problems, such as feeling depressed or anxious? | -1.54 |
| Overall, how satisfied have you been in the past month with the help that you have received from friends, neighbors, or family members for the activities that we just talked about? | -1.51, -0.11, 0.89 |
| During the past four weeks, were you limited in the kind of work or other activities you do as a result of your physical health? | -1.47 |
| ABLE: Do you use any kind of assistive device or special equipment (for example a lift chair, transfer board, or other seating adaptation) to help you move in and out of a chair? | -1.46 |
| walking a mile, taking rests as necessary | -1.43, -1.01, -0.59, -0.23 |
| Walking several blocks? | -1.40, -1.00, -0.63, -0.14 |
| taking a 1 mile, brisk walk without stopping to rest | -1.40, -1.00, -0.63, -0.14 |
| keeping in touch with others through letters, phone, or email | -1.40, -0.27, 1.59, 4.02 |
| COPE: Do you get help from another person to use the toilet, including getting on and off the toilet? | -1.35 |
| ALZQOL: Do you use any kind of assistive device or special equipment (for example a lift chair, transfer board, or other seating adaptation) to help you move in and out of a chair? | -1.32 |
| Do you use any kind of assistive device or special equipment (such as any seating adaptation) to help you get in and out of a car? | -1.31 |
| TAP: Do you get help from another person to use the toilet, including getting on and off the toilet? | -1.28 |
| During the past four weeks, have you accomplished less than you would like to as a result of your physical health? | -1.26 |
| Participating in strenuous activities or sports (such as running or tennis)? | -1.19, -0.66, -0.05, 0.84 |
| hiking a couple of miles on uneven surfaces, including hills | -1.12, -0.66, -0.19, 0.67 |
| ABLE: Do you use any kind of assistive device or special equipment (for example a lift chair, transfer board, or other seating adaptation) to help you move in and out of a chair? | -1.10 |
| taking care of your own health (eg medications) | -1.08, 0.16, 1.69, 4.08 |
| ABLE: Do you use any kind of assistive device or special equipment (for example a rocker knife, dycem, or any adapted utensils) to help you feed yourself? | -1.05 |
| How confident are you that you can find a way to use the telephone? | -1.04, -0.06, 1.37, 3.03 |
| How confident are you that you can find a way to take medication? | -1.01, 0.02 1.59, 2.85 |
| COPE: Do you use any kind of assistive device or special equipment (for example a rocker knife, dycem, or any adapted utensils) to help you feed yourself? | -1.01 |
| How confident are you that you can find a way to feed yourself? | -0.97, -0.12, 1.55, 4.13 |
| Do you use any kind of assistive device or special equipment (for example a hospital bed, rails, or bars) to help you move in and out of bed? | -0.96 |
| ALZQOL: Do you get help from another person to move in and out of a chair? | -0.96 |
| preparing meals for yourself | -0.95, 0.18, 1.96, 3.02 |
| COPE: Do you get help from another person to move in and out of a chair? | -0.95 |
| TAP: Do you get help from another person to move in and out of a chair? | -0.95 |
| ACT: Do you use any kind of assistive device or special equipment (for example a rocker knife, dycem, or any adapted utensils) to help you feed yourself? | -0.84 |
| COPE: Do you get help from another person to move in and out of a chair? | -0.78 |
| walking around one floor of your home, taking into consideration thresholds, doors, furniture, and a variety of floor coverings | -0.71, 0.70, 2.64 |
| COPE: Do you get help from another person to feed yourself? | -0.67 |
| In the past month, how difficult was it for you to use the telephone? | -0.59, 0.45, 1.52 |
| TAP: Do you get help from another person to move in and out of a chair? | -0.54 |
| ALZQOL: During the past four weeks, how much of the time has your physical health or emotional problems interfered with your social activities like visiting with friends, relatives, etc.? | -0.54, 0.76, 2.40, 4.51 |
| ALZQOL: Do you get help from another person to use the toilet, including getting on and off the toilet? | -0.52 |
| ALZQOL: Do you use any kind of assistive device or special equipment (for example grab bars, commode, or a raised toilet seat) to help you use the toilet, including getting on and off the toilet? | -0.52 |
| ABLE: Do you get help from another person to feed yourself? | -0.48 |
| COPE: Do you use any kind of assistive device or special equipment (for example a reacher, button hooks, velcro clothing) to help you dress above the waist? | -0.43 |
| ACT: Do you get help from another person to feed yourself? | -0.40 |
| ALZQOL: During the past four weeks, how much did pain interfere with your normal work, including both work outside the home and housework? Did it interfere? | -0.38, 0.83, 2.36, 4.38 |
| taking care of your own personal care needs | -0.37, 0.94, 2.87, 3.72 |
| REACH II: Do you get help from another person to feed yourself? | -0.35 |
| ABLE: Do you get help from another person to take medication? | -0.14 |
| ALZQOL: Do you use any kind of assistive device or special equipment (for example a lift chair, transfer board, or other seating adaptation) to help you move in and out of a chair? | -0.13 |
| ALZQOL: Do you get help from another person to move in and out of a chair? | -0.13 |
| Climbing several flights of stairs. Does your health now limit you a lot, limit you a little, or not limit you at all? | 0.15, 1.92 |
| holding a full glass of water in one hand | 0.43, 1.93, 3.19 |
| Do you get help from another person to walk one block? | 0.53 |
| First, consider moderate activities, such as moving a table, pushing a vacuum cleaner, bowling, or playing golf. Does your health now limit you a lot, limit you a little, or not limit you at all. | 0.53, 1.92 |
| Do you get help from another person to climb one flight of stairs? | 0.58 |
| ABLE: Do you use any kind of assistive device or special equipment (for example large handled items, adapted faucets) to help you groom, such as brushing teeth, combing or brushing hair, washing hands, washing face, and either shaving or applying makeup? | 0.66 |
| ABLE: Do you get help from another person to USE THE TELEPHONE? | 0.76 |
| Do you get help from another person to move in and out of bed? | 1.17 |
| Do you get help from another person to walk indoors, such as around your home? | 1.36 |
