# Supplementary material for: E pluribus unum: Harmonization of physical functioning across intervention studies of middle-aged and older adults
Source: PLoS One. 2017 Jul 28;12(7):e0181746. doi: 10.1371/journal.pone.0181746 (PMC5533461; doi:10.1371/journal.pone.0181746)
Supplement: S2 Table — * We used an age cutoff of 65 years in the BECT study but 80 in the ALZQOL study because of too few participants (N = 3) younger than 65 years in that study. ** Physical frailty was defined by Fried’s criteria: weight loss, exhaustion, physical activity, walk time, grip strength; Robust: 0 criteria met, Pre-frail: 1–2 criteria met, Frail: 3+ criteria met. (DOCX) [file pone.0181746.s003.docx]

**S2 Table. Convergent criterion validity of the physical functioning measure by sex and age**

|  | **Women** | |  | **Men** | |  | **Age≥65 *** | |  | **Age<65 *** | |
| --- | --- | --- | --- | --- | --- | --- | --- | --- | --- | --- | --- |
| **Objective Functioning Measures** | **Mean physical functioning score** | **p-value relative to reference** |  | **Mean physical functioning score** | **p-value relative to reference** |  | **Mean physical functioning score** | **p-value relative to reference** |  | **Mean physical functioning score** | **p-value relative to reference** |
| Timed Up and Go Task (ALZQOL) |  |  |  |  |  |  |  |  |  |  |  |
| Normal mobility (<20 seconds) | 41.1 | REF |  | 52.5 | REF |  | 43.9 | REF |  | 42.0 | REF |
| Problems (21-30 seconds) | 49.9 | <0.01 |  | 54.4 | 0.86 |  | 50.8 | <0.01 |  | 55.2 | <0.01 |
| Tandem balance (BECT) |  |  |  |  |  |  |  |  |  |  |  |
| Held 10+ seconds | 27.8 | REF |  | 25.4 | REF |  | 27.5 | REF |  | 25.9 | REF |
| Not held | 29.1 | 0.04 |  | 26.2 | 0.73 |  | 28.7 | 0.10 |  | 28.0 | 0.08 |
| Gait speed (BECT) |  |  |  |  |  |  |  |  |  |  |  |
| Faster than 0.8 meters/s | 27.6 | REF |  | 25.3 | REF |  | 25.8 | REF |  | 23.7 | REF |
| Slower than 0.8 meters/s | 29.9 | <0.01 |  | 27.9 | 0.18 |  | 27.6 | 0.01 |  | 27.1 | <0.01 |
| Chair stands, time to complete (BECT) | |  |  |  |  |  |  |  |  |  |  |
| Faster than median (12.6 meters/s) | 27.0 | REF |  | 23.1 | REF |  | 25.2 | REF |  | 22.3 | REF |
| Slower than median | 28.4 | 0.01 |  | 27.3 | <0.01 |  | 26.6 | 0.03 |  | 23.6 | <0.01 |
| Grip strength (BECT) |  |  |  |  |  |  |  |  |  |  |  |
| Stronger than median (52 lb) | 26.5 | REF |  | 25.0 | REF |  | 25.7 | REF |  | 23.8 | REF |
| Weaker than median | 29.2 | <0.01 |  | 26.4 | 0.28 |  | 27.6 | <0.01 |  | 27.1 | <0.01 |
| Physical frailty** (BECT) |  |  |  |  |  |  |  |  |  |  |  |
| Robust Status | 27.6 | REF |  | 24.0 | REF |  | 25.2 | REF |  | 25.1 | REF |
| Pre-frail/Frail Status | 29.4 | <0.01 |  | 28.6 | <0.01 |  | 27.4 | <0.01 |  | 27.2 | 0.02 |
|  |  |  |  |  |  |  |  |  |  |  |  |
| * We used an age cutoff of 65 years in the BECT study but 80 in the ALZQOL study because of too few participants (N=3) younger than 65 years in that study. | | | | | | | | | | | |
| ** Physical frailty was defined by Fried’s criteria: weight loss, exhaustion, physical activity, walk time, grip strength; Robust: 0 criteria met, Pre-frail: 1-2 criteria met, Frail: 3+ criteria met | | | | | | | | | | | |
